# Comparison of platinum monotherapy with concurrent chemoradiation therapy versus platinum-based dual drug therapy with concurrent chemoradiation therapy for locally advanced cervical cancer: a systematic review and meta-analysis

**DOI:** 10.1186/s13027-022-00433-3

**Published:** 2022-04-19

**Authors:** Ting Deng, Shequn Gu, Jianchi Wu, Yuanyi Yu

**Affiliations:** 1 Department of Gynecology, The First People’s Hospital of Chenzhou City, No.102, Luojiajing, Beihu District, Chenzhou City, 423000 Hunan Province China; 2 Department of Oncology, The First People’s Hospital of Chenzhou City, Chenzhou City, 423000 Hunan Province China

**Keywords:** Locally advanced cervical cancer, Chemotherapy, Concurrent chemoradiation therapy, Platinum, Meta-analysis

## Abstract

**Objective:**

To compare the survival outcomes and adverse events of patients with locally advanced cervical cancer (LACC) who received platinum monotherapy with concurrent chemoradiation therapy (CCRT) versus platinum-based dual drug therapy with CCRT.

**Method:**

All relevant literature was screened form the PubMed, EMBASE, Web of Science, The Cochrane Library and other databases from their establishment to October 2020. The main endpoint indicators included overall survival (OS) and progression-free survival (PFS). Grade 3 and above adverse events induced by chemotherapy were also compared.

**Results:**

This study involved 17 literature and 4,106 patients. There were 2,066 patients treated with CCRT with platinum-based dual drug therapy and 2,040 patients received CCRT with platinum monotherapy. Meta-analysis results showed that, compared to CCRT with platinum monotherapy, OS (HR = 0.68, 95% CI 0.58–0.79) and PFS (HR = 0.67, 95% CI 0.58–0.77) of LACC patients were significantly improved by CCRT with platinum-based dual drug therapy. In addition, CCRT with platinum-based dual drug therapy led to more adverse reactions such as neutropenia (OR = 4.92, 95% CI 3.55–6.84), anemia (OR = 1.99, 95% CI 1.17–3.39), diarrhea (OR = 1.70, 95% CI 1.30–2.22), leukopenia (OR = 2.42, 95%CI 1.84–3.17), thrombocytopenia (OR = 2.87, 95%CI 1.44–5.72), etc.

**Conclusion:**

CCRT with platinum-based dual drug therapy improved OS and PFS of LACC patients relative to the CCRT with platinum monotherapy. But it also increased the adverse reactions caused by multiple chemotherapy drugs. Thus, it is crucial to select a proper chemotherapy regimen based on the actual tolerance of patients in clinical practice.

## Introduction

Cervical cancer is the fourth most frequent cancer in women throughout the world, followed by lung cancer, colon cancer, and breast cancer [1]. Patients with cervical cancer are usually diagnosed in the advanced stage. Locally advanced cervical cancer (LACC) refers to local tumor with a diameter greater than 4 cm in the narrow sense and refers to stage IB2-IVA cervical cancer in the broad sense [2]. The latest National Comprehensive Cancer Network (NCCN) guidelines recommend concurrent chemoradiation therapy (CCRT) including external radiotherapy and brachytherapy, which is the standard treatment method for LACC patients [3, 4]. Although CCRT can reduce recurrence and improve survival, clinical outcomes in patients are far from satisfactory.

Several studies compared the efficacy of platinum-based chemotherapy combinations in advanced cervical cancer patients. For example, a phase III randomized controlled trial compares the efficacy and safety of cisplatin with or without S-1 in stage IVB, recurrent, or persistent cervical cancer patients, while S-1 plus cisplatin does not show superiority over cisplatin alone in OS but it remarkably increases PFS [5]. Another retrospective study compared the efficacy of paclitaxel/ifosfamide/platinum triplet and paclitaxel/cisplatin doublet on patients with metastatic, recurrent, or persistent cervical cancer, showing a higher response rate than paclitaxel/platinum without an increase in severe complications [6]. Therefore, it was speculated that platinum-based combination chemotherapy may be an effective measure for CCRT in LACC patients, though in some studies this therapy did not show an advantage. Combination chemotherapy with cisplatin and paclitaxel along with radiotherapy in LACC patients is well-tolerated, but it seems that no increase existed in tumor response and progression-free survival (PFS) [7]. It was also suggested that adding gemcitabine at the CCRT phase does not provide substantially superior results, but treatment toxicities may be increased [8]. Therefore, the survival benefit of combination chemotherapy remained to be confirmed. This study compared the efficacy and safety of CCRT with platinum-based dual drug therapy versus CCRT with platinum monotherapy in the treatment of LACC via systematic review and meta-analysis, so as to offer references for the treatment selection of LACC patients.

## Methods

### Literature retrieval

Current systematic reviews and meta-analyses followed Preferred Reporting Items for Systematic reviews and Meta-Analyses (PRIAMA) statement [9]. Relevant literature in PubMed, EMBASE, Web of Science, The Cochrane Library and other databases were searched from their establishment to October 2020, using the keywords: ‘cervical cancer’, ‘cervical carcinoma’, ‘uterine cervix cancer’, ‘concurrent chemoradiotherapy’, ‘CCRT’, ‘concurrent radiotherapy and chemotherapy’ and ‘chemoradiotherapy’ in the ‘title’ and ‘abstract’ section with Medical Subject Heading (MeSH) terms and their combinations. All retrieved literature and their references were reviewed to include all literature that might meet the requirements. The specific retrieval strategy was as follows: (((cervical[Title/Abstract]) OR (cervix*[Title/Abstract])) AND (((cancer*[Title/Abstract]) OR (carcinoma*[Title/Abstract])) OR (neoplasm*[Title/Abstract]))) AND (((Chemoradiotherap*[Title/Abstract]) OR (Radiochemotherap*[Title/Abstract])) AND (((Concurrent[Title/Abstract]) OR (Synchronous*[Title/Abstract])) OR (Concomitant*[Title/Abstract]))).

### Selection of literature

The relevant literature was selected based on the following criteria: (1) Subjects: patients were pathologically diagnosed with LACC (clinical stage IIB-IVA according to the International Federation of Gynecology and Obstetrics (FIGO)); (2) All the included studies were retrospective or prospective randomized controlled trials comparing CCRT with platinum-based dual drug therapy versus CCRT with platinum monotherapy; (3) The primary endpoint indicators included OS and PFS, and the secondary endpoint indicator included grade 3 and above adverse reactions caused by chemotherapy. Exclusion criteria: (1) Studies of patients (FIGO IB-IIB) receiving preoperative neoadjuvant chemoradiotherapy or postoperative adjuvant CCRT; (2) Patients with recurrences or distant metastases and patients with severe medical diseases (performance status (PS) ≥ 2); (3) Non-English literature or Chinese literature; (4) Comments, reviews, meta-analyses, case reports, letters, expert opinions, etc.

### Data extraction and quality assessment

Data were independently extracted by two investigators, and disputes were negotiated by a third investigator. Data were extracted from the literature as follows: author, year of publication, study type, FIGO stage, median age, treatment, and sample size. Primary endpoints included OS and PFS, while HR and 95% CI were extracted from complete OS curves or sufficient survival data. In addition, grade 3 and above chemotherapy-related adverse reactions were assessed. Literature quality was evaluated using the Cochrane bias risk assessment tool. The scale assessed the risk of bias for each included literature from the following 6 aspects with 7 items in total: selection (including random sequence and allocation concealment), implementation (double-blind), measuring (blind evaluation of the results), the follow-up (integrity of endpoint data), report (results of selective reports) and others (other bias sources). The results of “low risk bias”, “high risk bias” and “unclear” were made for each item based on the bias risk assessment criteria.

### Statistical analysis

The extracted data were statistically analyzed by Stata 14.0 software. The heterogeneity of the results was verified and assessed by X^2^ test and represented by I^2^ index or p-value. No significant heterogeneity was in the included studies when *P* > 0.1 or I^2^ < 50%, with application of the fixed-effect model. Otherwise, random-effect model was used. Subgroup analysis was performed to explain the underlying source of heterogeneity if significant heterogeneity occurred in the included studies. HR and 95% CI data were extracted from survival data (OS and PFS) to represent treatment effects. HR < 1 indicated a better survival in platinum doublets group. When HR was not provided in the original text, we used Engauge Digitizer 4.1 software to estimate HR from survival curves. OR was used to represent the aggregate outcome of adverse events. OR > 1 indicated that adverse events occurred more frequently in the CCRT with platinum-based dual drug therapy group. Additionally, publication bias was evaluated by visual funnel plot and Egger regression asymmetry test, and *P* < 0.05 was defined as statistically significant.

## Results

### Literature retrieval results

**Fig. 1:**
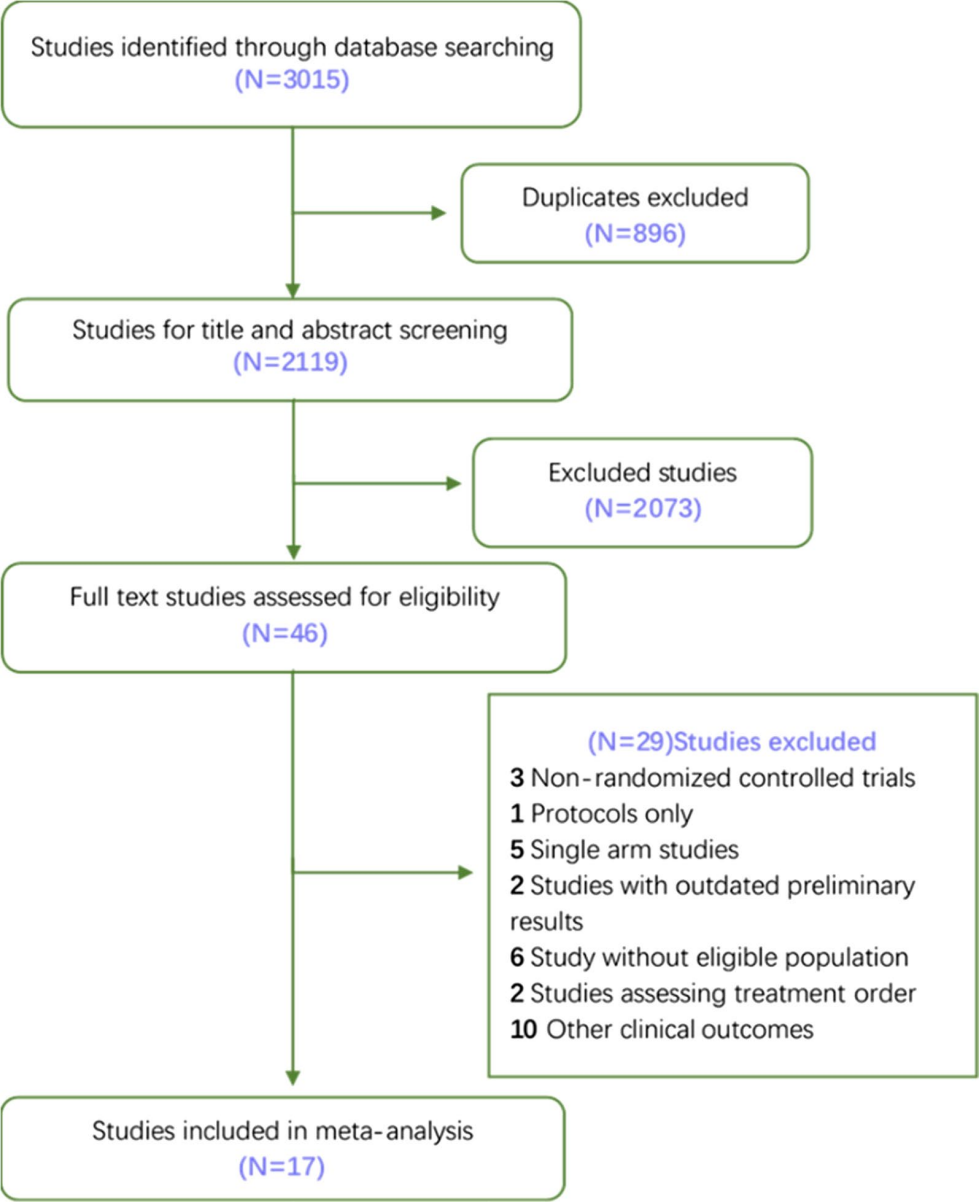
Flow of retrieving the literature

Through the retrieval strategy formulated above, a total of 3,015 literature was found. 896 duplicated literature was discarded and other 2,073 literature was removed by browsing titles and abstracts. Thus, 46 literature was selected for full-text paper review and 17 literature was included in this study. Screening process for the literature was shown in Fig. 1.

### Basic features of literature and results of quality assessment

**Fig. 2:**
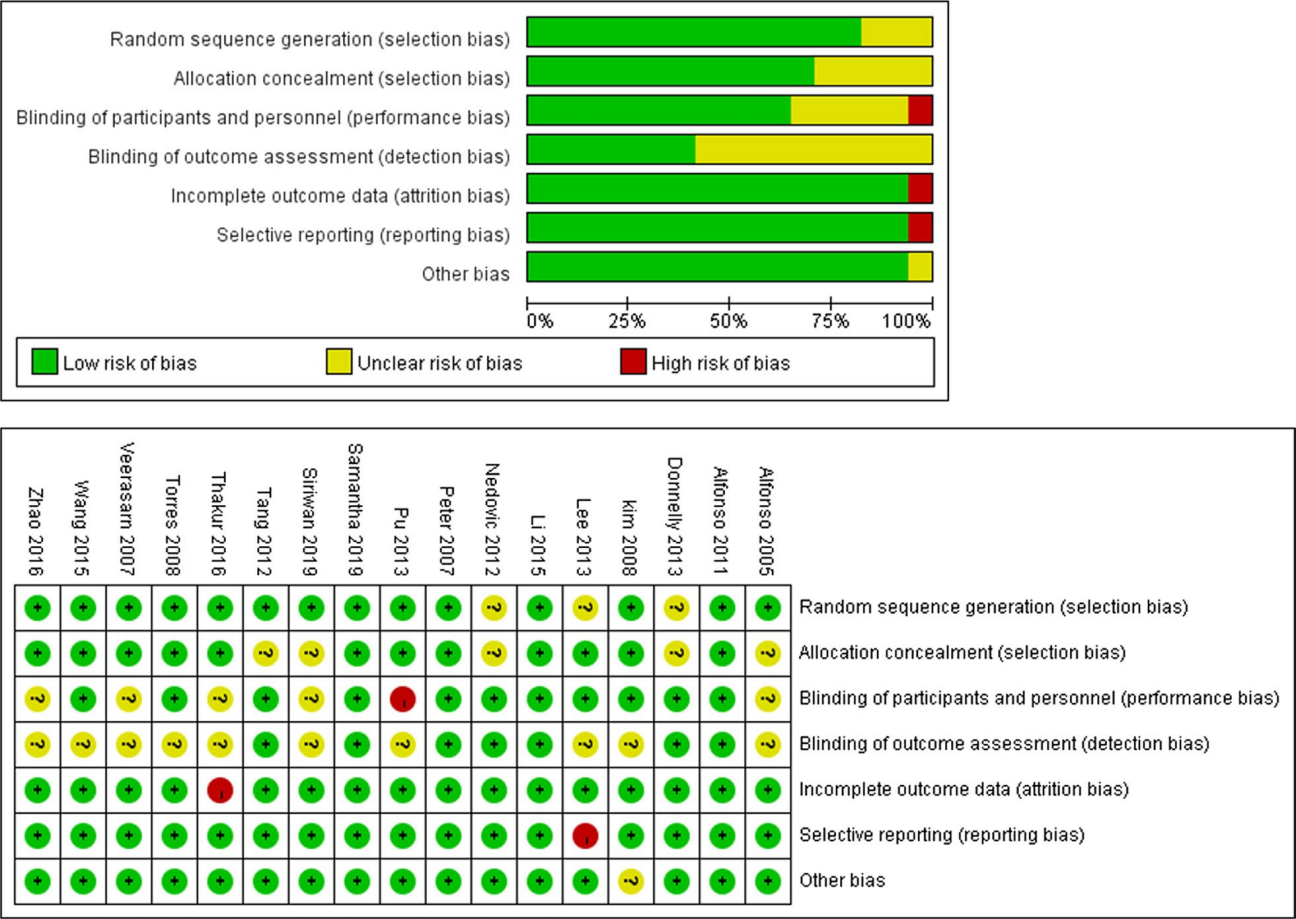
Results of Cochrane risk bias assessment

This study included 17 literature with 4,106 patients, while the literature consisted of 5 retrospective studies [10–14] and 12 prospective studies [4, 8, 15–24]. Among them, 2,066 patients received platinum-based dual drug therapy combining CCRT, while 2,040 patients received platinum monotherapy combining CCRT. Except for 5 retrospective studies, the rest were prospective studies. Basic features of the included literature were displayed in Table 1. The quality assessment results were shown in Fig. 2.

**Table 1:** The basic features of the included literature

Author	Year	Type of study	FIGO Stage	Median age (exp/ctr)	Patients (exp/ctr)	Treatment		HR (95% CI)	
						RT doses	CT regimens	OS	PFS
Alfonso [23]	2005	Prospective	IB2–IIB	41/49	43/40	EBRT 50 Gy + BT 30–35 Gy if high risk factors	Cisplatin + GEM/ cisplatin	0.18 (0.01–3.80)	0.17 (0.02–1.49)
Veerasarn [15]	2007	Prospective	IIB–IVA	49.6/49.7	234/235	EBRT 40–50 Gy/20–25 F + ICRT	Tegafur-uracil + carboplatin/carboplatin	0.90 (0.52–1.56)	
Peter [18]	2007	Prospective	IIB–III	NR	176/173	EBRT 40.8–51 Gy + BT 30–40 Gy	Cisplatin + 5-FU + HU/ cisplatin	0.90 (0.59–1.38)	0.99 (0.65–1.51)
Kim [21]	2008	Prospective	IIB–IVA	60/57	79/79	EBRT 41.4–50.4 Gy/23–28 F + ICRT 30–35 Gy/6–7 F	5-FU + cisplatin/ cisplatin	0.98 (0.47–2.04)	1.28 (0.68–2.41)
Torres [16]	2008	Prospective	III–IV	NR	191/111	EBRT 45 Gy (most pts) + LDR BT (most pts)	Cisplatin + 5-FU / cisplatin	0.42 (0.25–0.69)	
Alfonso [22]	2011	Prospective	IIB–IVA	45/46	259/256	EBRT 50.4 Gy/28 F + ICRT 30 Gy/6 F	GEM + cisplatin/ cisplatin	0.68 (0.49–0.95)	0.68 (0.49–0.95)
Nedovic [12]	2012	Retrospective	IIB–IVA	51/54	64/70	EBRT 50.4–54 Gy/20–30 F + ICRT 30–34 Gy/5 F	5-FU + cisplatin/ cisplatin	0.66 (0.34–1.28)	0.68 (0.37–0.99)
Tang [13]	2012	Retrospective	IIB–IVA	53/57	440/440	EBRT 48–50 Gy/24–25F + ICRT 24 Gy/4 F	PAC + cisplatin/ cisplatin	0.76 (0.56–1.04)	0.62 (0.48–0.80)
Donnelly [10]	2013	Retrospective	IB1–IVA	NR	42/95	EBRT 51.42 + LDR-BT	Cisplatin + 5-FU/ cisplatin	0.85 (0.38–1.92)	0.84 (0.39–1.83)
Lee [11]	2013	Retrospective	IB–IIA	45.5/44.5	21/34	EBRT 50.4 Gy (BT not done)	Cisplatin + CTX or cisplatin + 5-FU or carboplatin + 5-FU or carboplatin + PAC/ cisplatin	0.78 (0.17–3.51)	0.26 (0.07–0.95)
Pu [19]	2013	Prospective	IB–IIA	47/45	145/140	EBRT 46–54 Gy + BT 24 Gy	Cisplatin + DOC/ cisplatin	0.65 (0.39–1.09)	0.64 (0.40–1.03)
Wang [8]	2015	Prospective	III–IVA	55/56	37/37	EBRT 45 Gy/25 F + ICRT 25.8 Gy/6 F	GEM + cisplatin/ cisplatin	0.93 (0.35–2.47)	0.86 (0.39–1.91)
Li [20]	2015	Prospective	IIB–IVA	51.7/49.8	36/36	EBRT 50 Gy/25 F + ICRT 10 Gy/2 F	S-1 + cisplatin/ cisplatin	0.86 (0.32–2.31)	0.89 (0.28–2.71)
Thakur [17]	2016	Prospective	IIA–IIIB	NR	39/42	EBRT 50 Gy/25 F + ICRT 10 Gy/3 F	PAC + cisplatin/ cisplatin	0.54 (0.18–1.61)	0.47 (0.20–1.09)
Zhao [14]	2016	Retrospective	IA2–MIIB	50/52	75/71	EBRT 46–50 Gy/23–25 F + BT 30 Gy/ 5F	PAC + cisplatin consolidation/PAC + cisplatin	0.70 (0.31–1.60)	0.80 (0.38–1.67)
Samantha [24]	2019	Prospective	IIB–IVA	48/45	55/52	EBRT 45 Gy/25–28 F + BT 28–30 Gy/4–5 F	Cisplatin + GEM/ cisplatin	2.79 (1.29–6.01)	1.84 (1.04–3.26)
Siriwan [4]	2019	Prospective	IIB–IVA	49/50	130/129	EBRT 54 Gy + ICRT 28 Gy	Cisplatin + carboplatin + PAC/ cisplatin	1.42 (0.81–2.49)	1.26 (0.82–1.96)

*NR* not reported, *exp* experimental group, *ctr* control group, *CT* chemical therapy, *RT* radiotherapy, *EBRT* external beam radiotherapy, *ICRT* intracavitary radiotherapy, *BT* brachytherapy, *LDR* low dose rate, *HU* hydroxyurea, *5-FU* 5-Fluorouracil, *GEM* Gemcitabine, *DOC* docetaxel, *PAC* paclitaxel, *CTX* cyclophosphamide

### Results of meta-analysis

#### Evaluation of OS and PFS

**Fig. 3:**
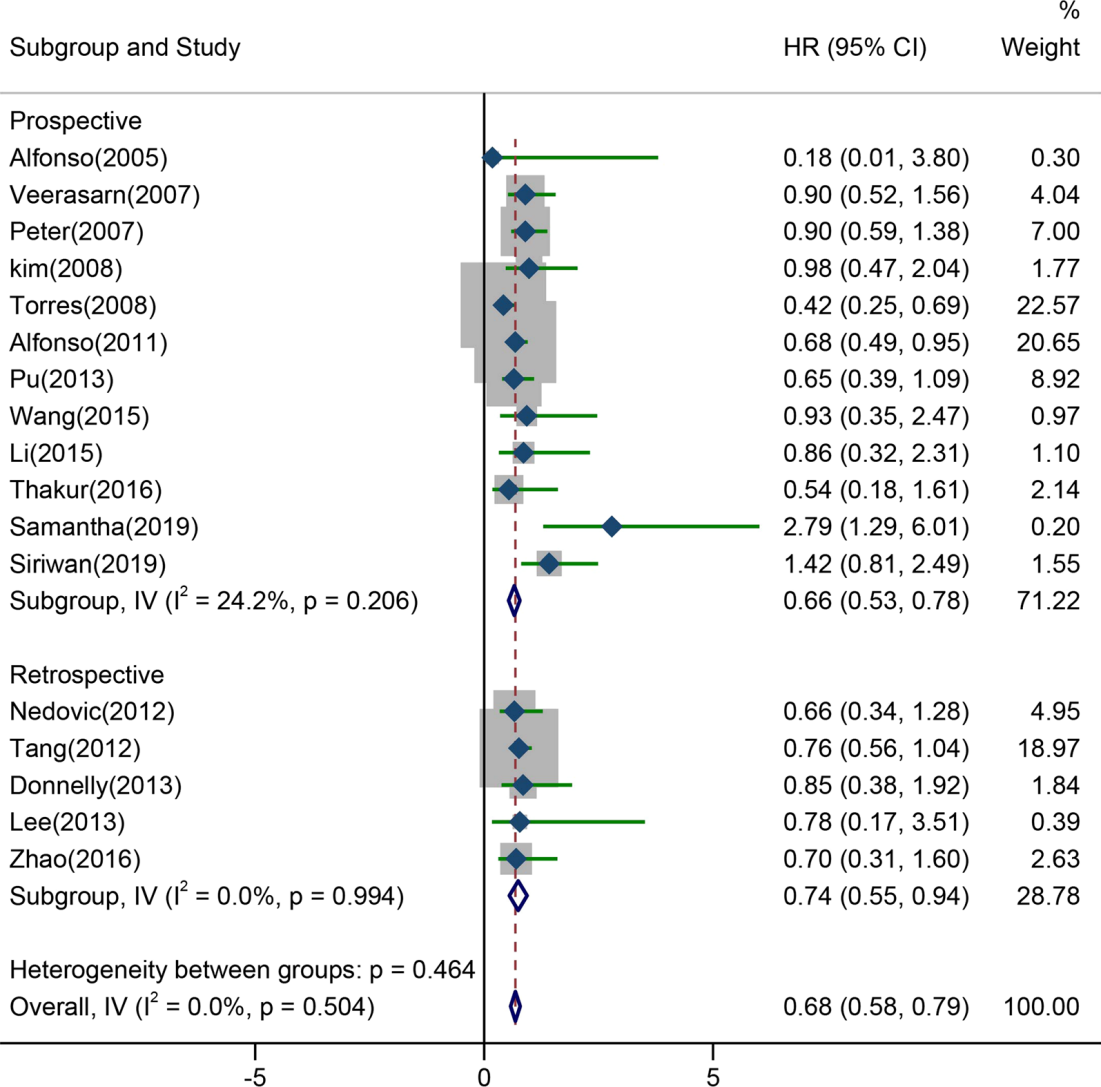
Forest plot of OS pooled results

The results of OS were reported in 17 studies, comprising 5 retrospective studies and 12 prospective studies. Since there was no heterogeneity (I^2^ = 0.0%, *P* = 0.504) confirmed through these studies, we used a fixed-effect model for analysis. The results of meta-analysis showed that CCRT with platinum-based dual drug therapy notably improved OS of LACC patients (HR = 0.68, 95% CI 0.58–0.79). The subgroup analysis on prospective studies exhibited that patients received platinum-based dual drug therapy combining CCRT had long OS (HR = 0.66, 95% CI 0.53–0.78), as displayed in Fig. 3.

**Fig. 4:**
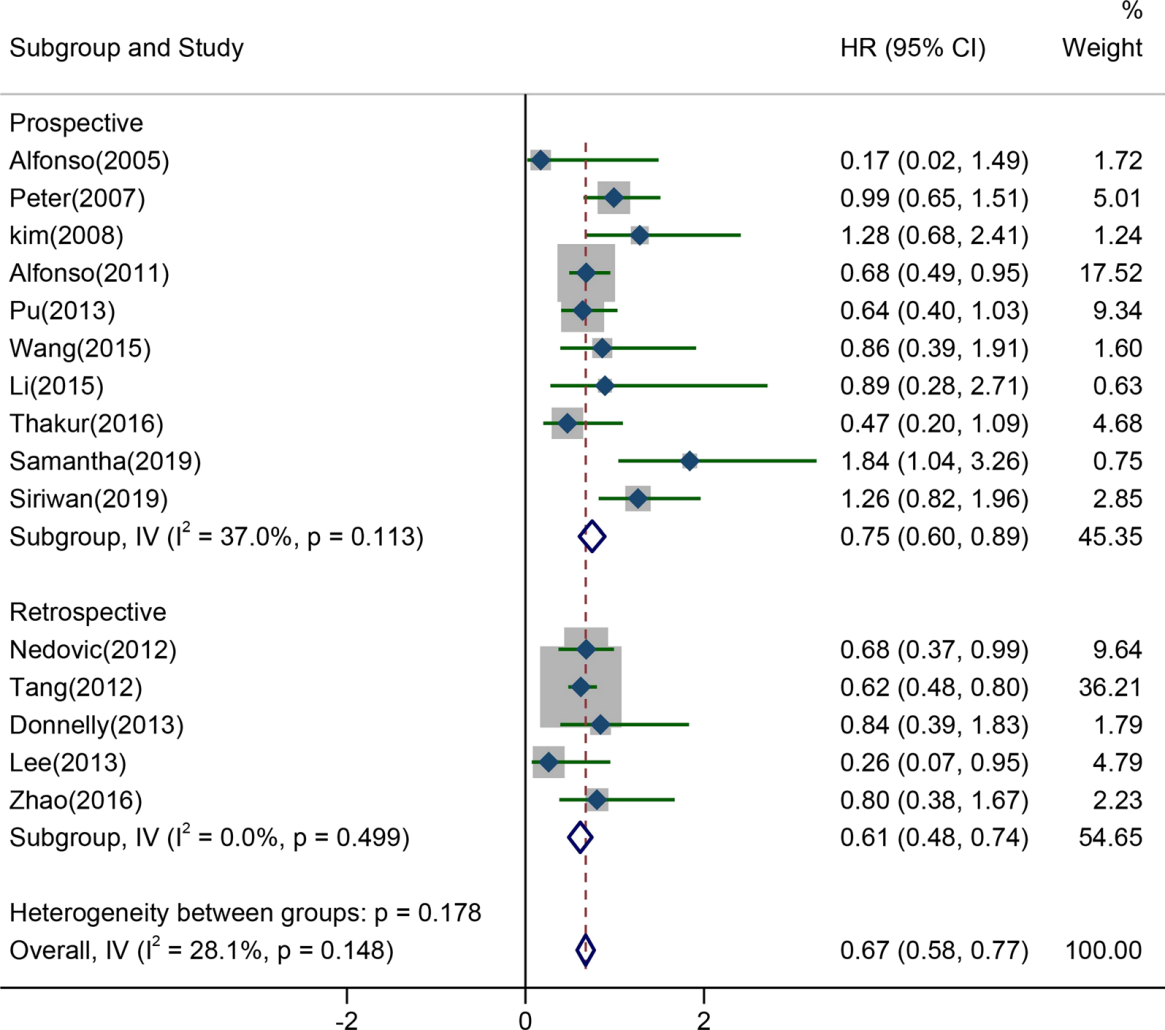
Forest plot of PFS pooled results

The results of PFS were reported in 15 studies, containing 5 retrospective studies and 10 prospective studies, showing low heterogeneity (I^2^ = 28.1%, *P* = 0.148). The results of meta-analysis showed that CCRT with platinum-based dual drug therapy remarkably prolonged PFS (HR = 0.67, 95% CI 0.58–0.77). The subgroup analysis on prospective studies illustrated that platinum-based dual drug therapy combining CCRT ameliorated patients’ PFS (HR = 0.75, 95% CI 0.60–0.89), as represented in Fig. 4. In conclusion, CCRT with platinum-based dual drug therapy presented more significant efficacy and significantly prolonged the survival of LACC patients, which had a broad application prospect.

#### Analysis of adverse events

**Fig. 5:**
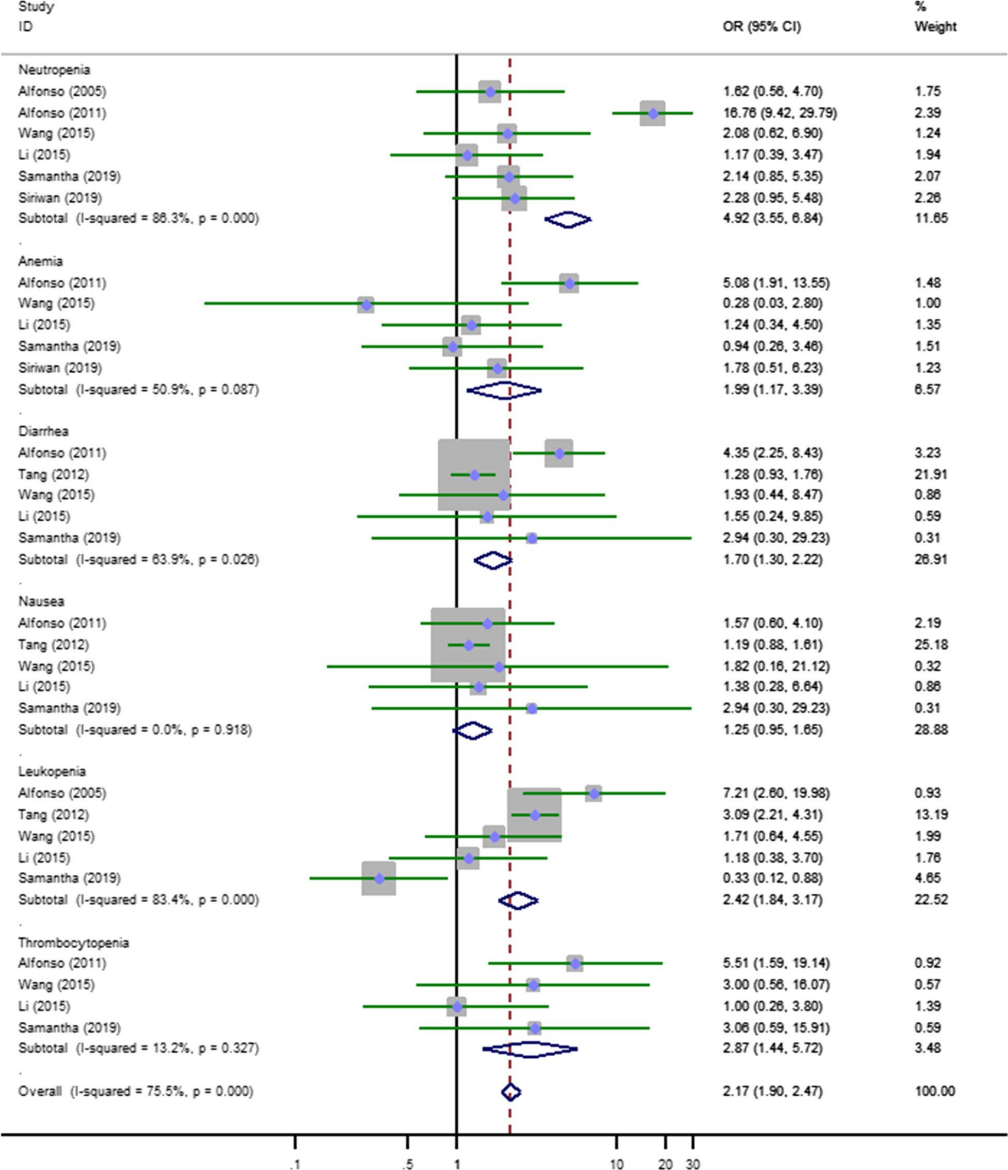
Forest plot of pooled results of adverse events

In addition, we performed a pooled analysis of grade 3 and above chemotherapy-related adverse events (Fig. 5). There were prominent differences in adverse events except for vomiting (OR = 1.25, 95% CI 0.95–1.65). Neutropenia (OR = 4.92, 95% CI 3.55–6.84), anemia (OR = 1.99, 95% CI 1.17–3.39), diarrhea (OR = 1.70, 95% CI 1.30–2.22), leukopenia (OR = 2.42, 95% CI 1.84–3.17), thrombocytopenia (OR = 2.87, 95% CI 1.44–5.72), etc. were significantly increased in the CCRT with platinum-based dual drug therapy group. It was obvious that multiple chemotherapeutic drugs would increase the occurrence of adverse events. Thus, the advantages and disadvantages should be weighed to maximize the survival of patients.

### Publication bias

**Fig. 6:**
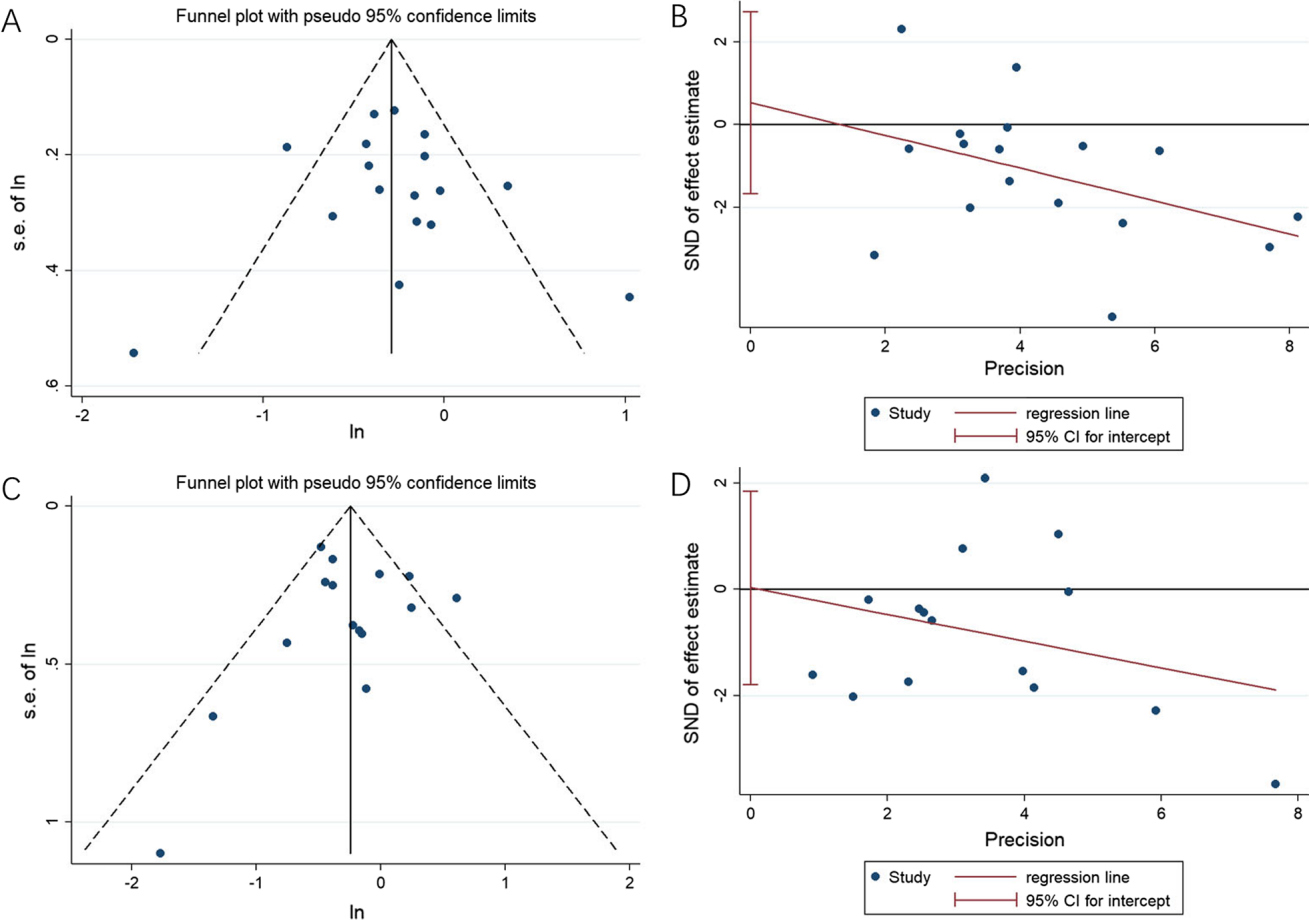
Publication bias. **A** OS of Begg’s test. **B** OS of Egger’s test. **C** PFS of Begg’s test. **D** PFS of Egger’s test

It was found that the funnel plot was basically symmetrical by observing visually. Assessed by Egger’s test (OS, *P* = 0.611; PFS, *P* = 0973), publication bias was not existed (Fig. 6). The evidence indicated that the included studies had no effect on the results of the meta-analysis.

## Discussion

CCRT is used as a standard nursing regimen for LACC patients, but whether CCRT with platinum-based dual drug therapy is more effective than the CCRT with platinum monotherapy needs to be further analyzed. It was indicated that CCRT with platinum-based dual drug therapy significantly improved OS and PFS of patients. Meanwhile, multiple combinations of chemotherapeutic drugs increased the adverse events caused by chemotherapy.

The main difference in efficacy is the combination of chemotherapy drugs. Cisplatin and carboplatin are two platinum drugs commonly used in chemotherapy. It is investigated that the main role of chemotherapy in CCRT is to enhance the sensitivity of radiotherapy (RT), and carboplatin concomitant with RT has similar efficacy and safety with single cisplatin [25, 26]. Similarly, another study confirmed this conclusion. The combined chemotherapy regimen is usually platinum chemicals combining docetaxel, paclitaxel, gemcitabine, fluorouracil, etc. A previous meta-analysis demonstrated the efficacy and safety of different concomitant chemotherapy in LACC patients and showed that CCRT (cisplatin + docetaxel) may be the optimal choice of CCRT regimens for LACC patients [27]. Notably, a retrospective study indicated that elderly LACC patients are well tolerated and achieved favorable survival outcomes after CCRT with platinum doublets agent [28]. Of course, differences in radiation intensity in each study cannot be ruled out. For example, intensity-modulated radiotherapy (IMRT) for cervical cancer can significantly reduce the incidence of acute enteritis, while standard three-dimensional conformal radiotherapy (3D CRT) shows no conspicuous difference in overall and disease-free survival [29]. A recent meta-analysis also compared the treatment outcomes of RT with CCRT with platinum-based doublets versus RT plus platinum single-agent therapy in LACC patients. The results showed that under the premise of good tolerance, LACC patients’ survival was extended via treating with RT plus platinum-based doublet therapy improves survival compared to RT plus platinum single-agent therapy [30], which were the same as our results. In addition, a large amount of recent literature was included in our study to ensure the robustness of the results. 5 retrospective studies and 12 prospective studies constituted the 17 included literature, which, associating with the further subgroup analysis on the prospective studies, suggested that CCRT with platinum-based dual drug therapy prolonged the OS and PFS of patients.

There are some limitations here. Firstly, we did not analyze the prognostic results of different types of cervical cancer. It was demonstrated that CCRT plays a vital role in treatment of LACC [31]. Secondly, in the included clinical trials, the chemotherapy schedules and timetable of patients were significantly different. As shown previously, paclitaxel plus cisplatin is the best chemotherapy regimen for cervical cancer [32]. Subsequently, the results of a recent phase III trial indicated that carboplatin-based regimen has similar efficacy with lower toxicity compared to cisplatin. Thus, carboplatin can be a standard treatment option instead of cisplatin [33]. Only two studies based on carboplatin doublets were included in our meta-analysis. Therefore, more clinical studies are needed to confirm the specific efficacy of these two platinum drugs in CCRT for patients with LACC.

In conclusion, CCRT with platinum-based dual drug therapy significantly improved the survival but increased adverse events of LACC patients compared to CCRT with platinum monotherapy. Thus, the actual tolerance of patients should be considered when choosing the best regimen.

## Data Availability

The data used to support the findings of this study are included within the article.

## References

[1] Bray F (2018). Global cancer statistics 2018: GLOBOCAN estimates of incidence and mortality worldwide for 36 cancers in 185 countries. CA Cancer J Clin.

[2] Zhao YB, Wang JH, Chen XX, Wu YZ, Wu Q (2012). Values of three different preoperative regimens in comprehensive treatment for young patients with stage Ib2 cervical cancer. Asian Pac J Cancer Prev.

[3] Koh WJ (2019). Cervical Cancer, Version 3.2019, NCCN clinical practice guidelines in oncology. J Natl Compr Canc Netw.

[4] Tangjitgamol S (2019). A randomized controlled trial comparing concurrent chemoradiation versus concurrent chemoradiation followed by adjuvant chemotherapy in locally advanced cervical cancer patients: ACTLACC trial. J Gynecol Oncol.

[5] Aoki Y (2018). Phase III study of cisplatin with or without S-1 in patients with stage IVB, recurrent, or persistent cervical cancer. Br J Cancer.

[6] Choi HJ (2018). Response to combination chemotherapy with Paclitaxel/Ifosfamide/Platinum versus paclitaxel/platinum for patients with metastatic, recurrent, or persistent carcinoma of the uterine cervix: a retrospective analysis. Int J Gynecol Cancer.

[7] Kalaghchi B, Abdi R, Amouzegar-Hashemi F, Esmati E, Alikhasi A (2016). Concurrent chemoradiation with weekly paclitaxel and cisplatin for locally advanced cervical cancer. Asian Pac J Cancer Prev.

[8] Wang CC (2015). A randomized trial comparing concurrent chemoradiotherapy with single-agent cisplatin versus cisplatin plus gemcitabine in patients with advanced cervical cancer: An Asian Gynecologic Oncology Group study. Gynecol Oncol.

[9] Liberati A (2009). The PRISMA statement for reporting systematic reviews and meta-analyses of studies that evaluate health care interventions: explanation and elaboration. PLoS Med.

[10] Donnelly ED (2015). Evaluation of outcomes in patients with carcinoma of the cervix treated with concurrent radiation and cisplatin versus cisplatin/5-FU compared with radiation alone. Am J Clin Oncol.

[11] Lee YY (2013). Platinum-based combination chemotherapy vs. weekly cisplatin during adjuvant CCRT in early cervical cancer with pelvic LN metastasis. Anticancer Res.

[12] Nedovic J (2012). Cisplatin monotherapy with concurrent radiotherapy versus combination of cisplatin and 5-fluorouracil chemotherapy with concurrent radiotherapy in patients with locoregionally advanced cervical carcinoma. J BUON.

[13] Tang J, Tang Y, Yang J, Huang S (2012). Chemoradiation and adjuvant chemotherapy in advanced cervical adenocarcinoma. Gynecol Oncol.

[14] Zhao H (2016). Concurrent paclitaxel/cisplatin chemoradiotherapy with or without consolidation chemotherapy in high-risk early-stage cervical cancer patients following radical hysterectomy: preliminary results of a phase III randomized study. Oncotarget.

[15] Veerasarn V (2007). A randomized phase III trial of concurrent chemoradiotherapy in locally advanced cervical cancer: preliminary results. Gynecol Oncol.

[16] Torres MA (2008). Comparison of treatment tolerance and outcomes in patients with cervical cancer treated with concurrent chemoradiotherapy in a prospective randomized trial or with standard treatment. Int J Radiat Oncol Biol Phys.

[17] Thakur P, Seam R, Gupta M, Gupta M (2016). Prospective randomized study comparing concomitant chemoradiotherapy using weekly cisplatin & paclitaxel versus weekly cisplatin in locally advanced carcinoma cervix. Ann Transl Med.

[18] Rose PG (2007). Long-term follow-up of a randomized trial comparing concurrent single agent cisplatin, cisplatin-based combination chemotherapy, or hydroxyurea during pelvic irradiation for locally advanced cervical cancer: a Gynecologic Oncology Group Study. J Clin Oncol.

[19] Pu J (2013). A randomized controlled study of single-agent cisplatin and radiotherapy versus docetaxel/cisplatin and radiotherapy in high-risk early-stage cervical cancer after radical surgery. J Cancer Res Clin Oncol.

[20] Li Z, Mao W, Lin N, Han S (2016). Concurrent radiotherapy with S-1 plus cisplatin versus concurrent radiotherapy with cisplatin alone for the treatment of locally advanced cervical carcinoma: a pilot randomised controlled trial. Clin Transl Oncol.

[21] Kim YS (2008). Prospective randomized comparison of monthly fluorouracil and cisplatin versus weekly cisplatin concurrent with pelvic radiotherapy and high-dose rate brachytherapy for locally advanced cervical cancer. Gynecol Oncol.

[22] Duenas-Gonzalez A (2011). Phase III, open-label, randomized study comparing concurrent gemcitabine plus cisplatin and radiation followed by adjuvant gemcitabine and cisplatin versus concurrent cisplatin and radiation in patients with stage IIB to IVA carcinoma of the cervix. J Clin Oncol.

[23] Duenas-Gonzalez A (2005). Pathologic response and toxicity assessment of chemoradiotherapy with cisplatin versus cisplatin plus gemcitabine in cervical cancer: a randomized Phase II study. Int J Radiat Oncol Biol Phys.

[24] da Costa SCS (2019). Neoadjuvant chemotherapy with cisplatin and gemcitabine followed by chemoradiation versus chemoradiation for locally advanced cervical cancer: a randomized phase II trial. J Clin Oncol.

[25] Nam EJ (2013). Comparison of carboplatin- and cisplatin-based concurrent chemoradiotherapy in locally advanced cervical cancer patients with morbidity risks. Oncologist.

[26] Sebastiao AM (2016). Carboplatin-based chemoradiotherapy in advanced cervical cancer: an alternative to cisplatin-based regimen?. Eur J Obstet Gynecol Reprod Biol.

[27] Fu ZZ (2017). Efficacy and toxicity of different concurrent chemoradiotherapy regimens in the treatment of advanced cervical cancer: a network meta-analysis. Medicine.

[28] Wang W (2017). Outcome and toxicity of radical radiotherapy or concurrent Chemoradiotherapy for elderly cervical cancer women. BMC Cancer.

[29] Yu C (2015). A comparative study of intensity-modulated radiotherapy and standard radiation field with concurrent chemotherapy for local advanced cervical cancer. Eur J Gynaecol Oncol.

[30] Ma S (2019). Platinum single-agent vs. platinum-based doublet agent concurrent chemoradiotherapy for locally advanced cervical cancer: a meta-analysis of randomized controlled trials. Gynecol Oncol.

[31] Kang JH (2020). Prognostic significance of tumor regression rate during concurrent chemoradiotherapy in locally advanced cervix cancer: analysis by radiation phase and histologic type. J Clin Med..

[32] Monk BJ (2009). Phase III trial of four cisplatin-containing doublet combinations in stage IVB, recurrent, or persistent cervical carcinoma: a Gynecologic Oncology Group study. J Clin Oncol.

[33] Kitagawa R (2015). Paclitaxel plus carboplatin versus paclitaxel plus cisplatin in metastatic or recurrent cervical cancer: the open-label randomized phase III trial JCOG0505. J Clin Oncol.

